# Supplementary material for: A Fluorescence Sensing Determination of 2,4,6-Trinitrophenol Based on Cationic Water-Soluble Pillar[6]arene Graphene Nanocomposite
Source: Sensors (Basel). 2018 Dec 28;19(1):91. doi: 10.3390/s19010091 (PMC6338956; doi:10.3390/s19010091)
Supplement: Supplementary file 1 [file sensors-19-00091-s001.pdf]

Suppporting Information

A fluorescence sensing determination of 2, 4, 6-trinitrophenol based on cationic water-soluble pillar[6]arene graphene nanocomposite

Xiaoping Tan*, Tingying Zhang, Wenjie Zeng, Shuhua He, Xi Liu, Hexiang Tian, Jianwei Shi, Tuanwu Cao*

Key Lab of Inorganic Special Functional Materials, Chongqing Municipal Education Commission, School of Chemistry and Chemical Engineering, Yangtze Normal University, Chongqing, 408100, China

S1. Reagents and methods

Hydroquinone, 1,2-dihydroxybenzene, boron trifluoride diethyl etherate, chloroform, carbon tetrabromide, triphenylphosphine, acetonitrile, paraformaldehyde, pyridine were reagent grade and used as received. Solvents were either employed as purchased or dried according to procedures described in the literature. ^{1}H NMR and ^{13}C NMR spectra were recorded on a Bruker Avance DMX-400 spectrometer at 400 MHz and 500 MHz. PCP6 [1-3] was synthesized according to the previous papers procedures.

S2. Synthesis of pyridine functionalized pillar[6]arene (PCP6)

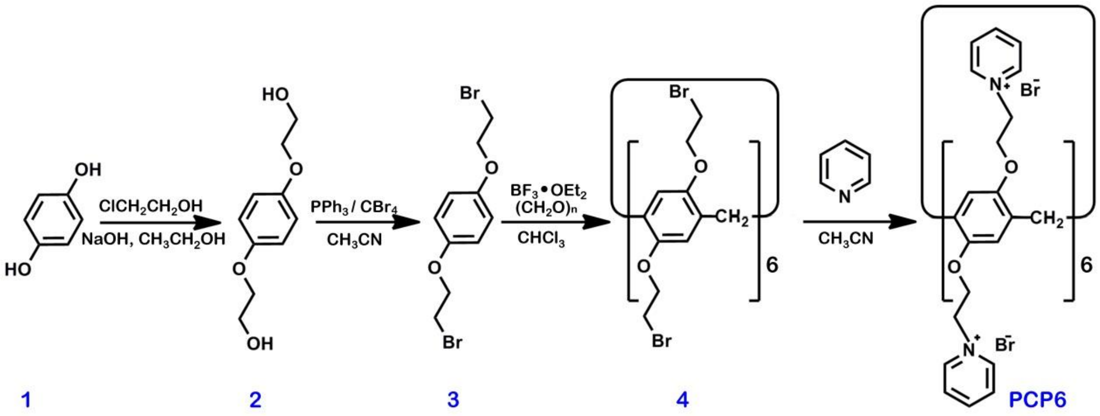

Scheme S1. Synthetic route of **PCP6**.

Synthesis of 2: 50 mL of aqueous solution of NaOH (12.5 g, 311.2 mmol) was added to 150 mL of ethanol solution containing 1,2-dihydroxybenzene (HQ, 11.43 g, 104.0 mmol) under stirring for 25 min followed by addition of 2-chloroethanol (25.1 g, 311.7 mmol) under refluxing for 72 h. After that, the pH of the mixture solution was adjusted to 6−7, and the solvent was removed by reduced pressure distillation. Ethanol (120 mL) was added to dissolve the residual solid at the elevated temperature, and then the hot mixture solution was filtered to remove insoluble inorganic salts. The filtrate was concentrated to get the crude product. The pure product **2** was obtained by recrystallization in ethanol. The ^{1}H NMR spectrum of **2** is shown in Figure S1. ^{1}H NMR (400 MHz, D_2O, rt) δ (ppm): 6.925 (s, 4H), 4.028 (t, *J* = 4.4 Hz, 4H), 3.821 (t, *J*

= 4 Hz, 4H). The ^{13}C NMR spectrum of **2** is shown in Figure S2. ^{13}C NMR (100 MHz, D_2O, rt) δ (ppm): 152.72, 116.24, 70.08, 60.15.

Synthesis of 3: A solution of **2** (10.0 g, 50.4 mmol) and triphenylphosphine (32.0 g, 120 mmol) in dry acetonitrile (250 mL) was cooled with an ice bath. Under vigorous stirring, carbon tetrabromide (40.0 g, 120 mmol) was slowly added. The mixture was stirred at room temperature for 4 hours. Then cold water (250 mL) was added to the reaction mixture to give white precipitation. The precipitate was collected, washed with methanol/water (3:2, 3 × 100 mL), recrystallized from methanol, and dried under vacuum to afford **C2** as white crystals (14.4 g, 92%). The ^{1}H NMR spectrum of **3** is shown in Figure S3. ^{1}H NMR (400 MHz, $CDCl_3$, rt) *δ* (ppm): 6.862 (s, 4H), 4.245 (t, *J* = 6 Hz, 4H), 3.616 (t, *J* = 6.4 Hz, 4H). The ^{13}C NMR spectrum of **3** is shown in Figure S4. ^{13}C NMR (100 MHz, $CDCl_3$, rt) δ (ppm): 152.8, 116.06, 68.68, 29.28.

Synthesis of 4: Boron trifluoride diethyl etherate (BF_3 OEt_2, 3.26 g, 23.0 mmol) was added to the mixed solution of paraformaldehyde (0.7 g, 23.0 mmol) and **3** (3.37 g, 11.5 mmol) in chloroform (200 mL) under nitrogen atmosphere. Then the mixture was stirred at room temperature for 3 hour. A green solution was obtained. The reaction mixture was then washed with water (3 × 120 mL) and dried with excess Na_2SO_4. After the solvent was removed, the obtained solid was purified by column chromatography on silica gel with petroleum ether/dichloromethane (1:2 *v*/*v*) as the eluent to get a white powder of **4** (0.52 g, 15 %). The ^{1}H NMR spectrum of **4** is shown in Figure S5. ^{1}H NMR (500 MHz, $CDCl_3$, rt) *δ* (ppm): 6.777 (s, 12H), 4.16 (t, *J* = 6 Hz, 24H), 3.867 (s, 12H), 3.545 (t, *J* = 6 Hz, 24H). The ^{13}C NMR spectrum of **C3** is shown in Figure S6. ^{13}C NMR (100 MHz, $CDCl_3$, rt) *δ* (ppm): 150.16, 128.51, 115.83, 68.95, 30.63, 30.32.

Synthesis of PCP6: An excess amount of pyridine (2.4 g, 30 mmol) was added to a solution of **4** (1.5 g, 0.74 mmol) in CH_3CN (50 mL), and the resulting mixture was refluxed for 30 hours. After cooling to room temperature, the precipitate was filtered, and was washed by CH_3CN and dried to give a light yellow solid **PCP6**. The ^{1}H NMR spectrum of **PCP6** is shown in Figure S7. ^{1}H NMR (500 MHz, DMSO-*d6*, rt) *δ* (ppm): 9.307 (s, 24H), 8.574 (t, *J=7.5* Hz, 12H), 8.057 (s, 24H), 6.549 (s, 12H), 5.145

(s, 24H), 4.474 (s, 24H), 3.328 (s, 12H). The ^{13}C NMR spectrum of **PCP6** is shown in Figure S8. ^{13}C NMR (125 MHz, DMSO-*d6*, rt) δ (ppm): 149.29, 145.91, 145.15, 127.66, 127.39, 115.30, 67.38, 60.09.

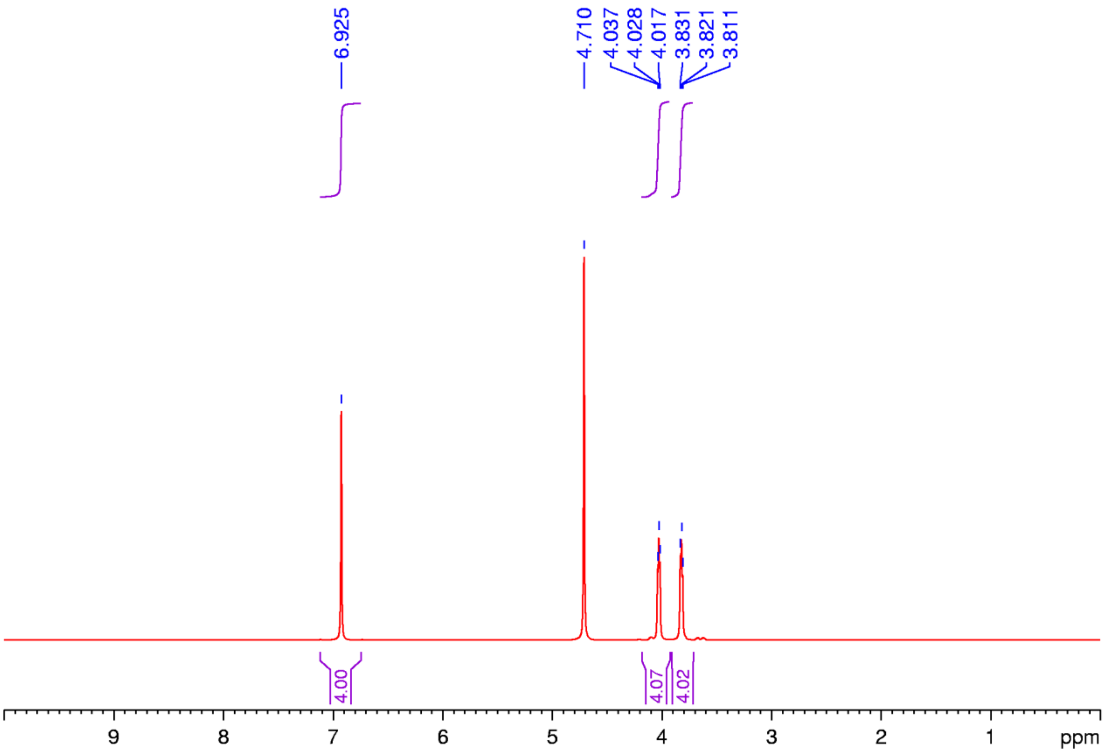

Figure S1. ^{1}H NMR spectrum (400 MHz, D_2O, 298 K) of **2**.

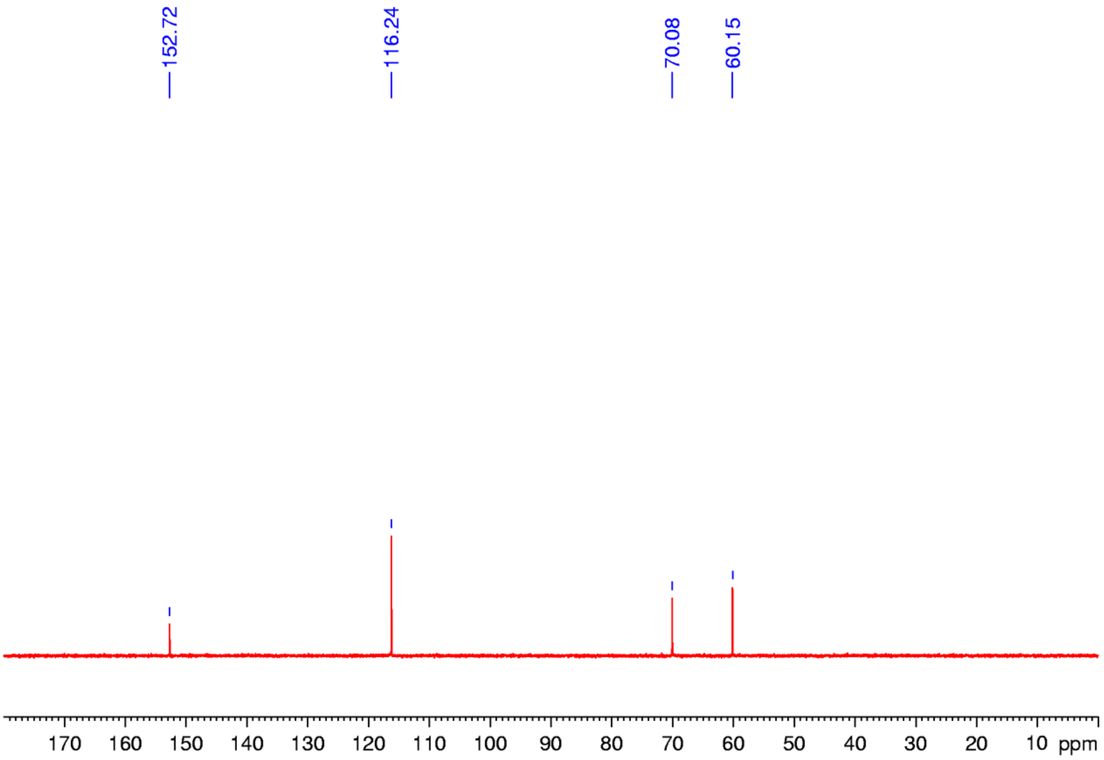

Figure S2. ^{13}C NMR spectrum (100 MHz, D_2O, 298 K) of **2**.

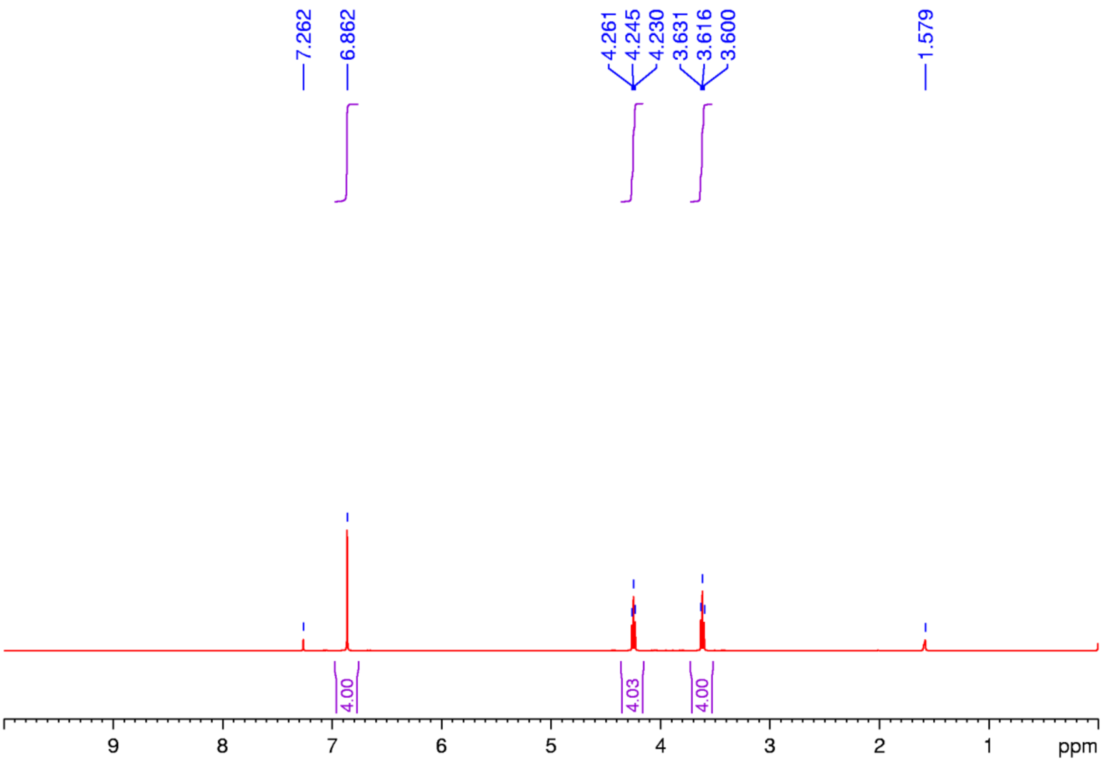

Figure S3. ^{1}H NMR spectrum (400 MHz, $CDCl_3$, 298 K) of **3**.

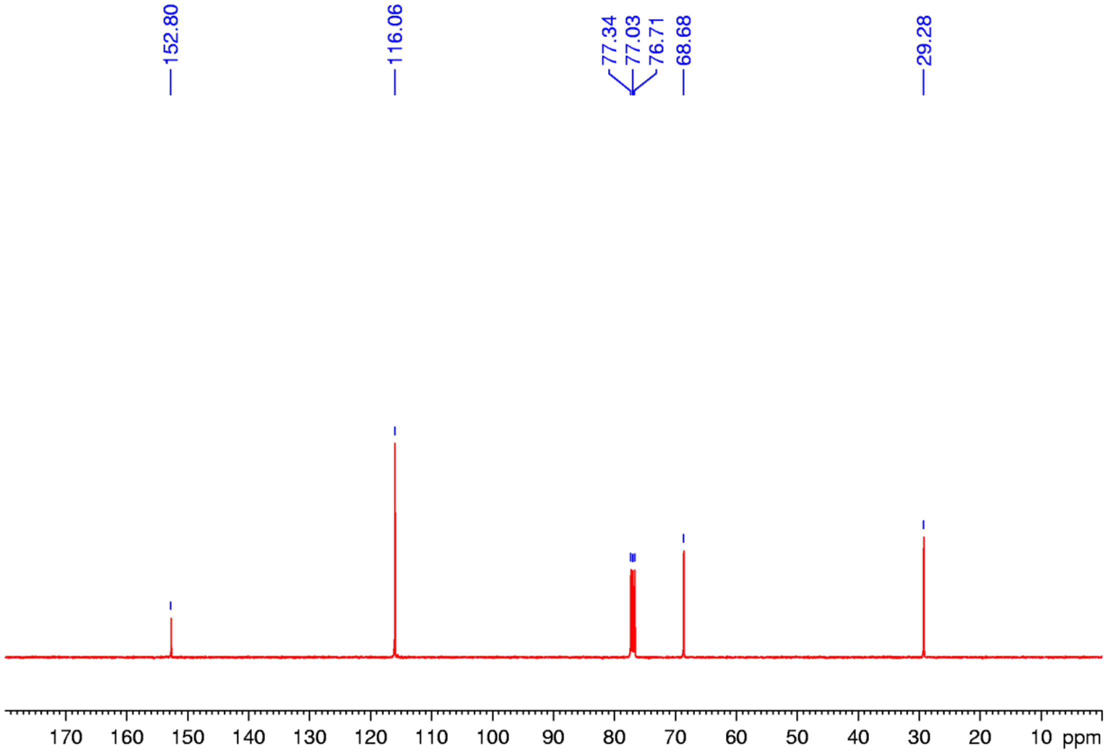

Figure S4. ^{13}C NMR spectrum (100 MHz, $CDCl_3$, 298 K) of **3**.

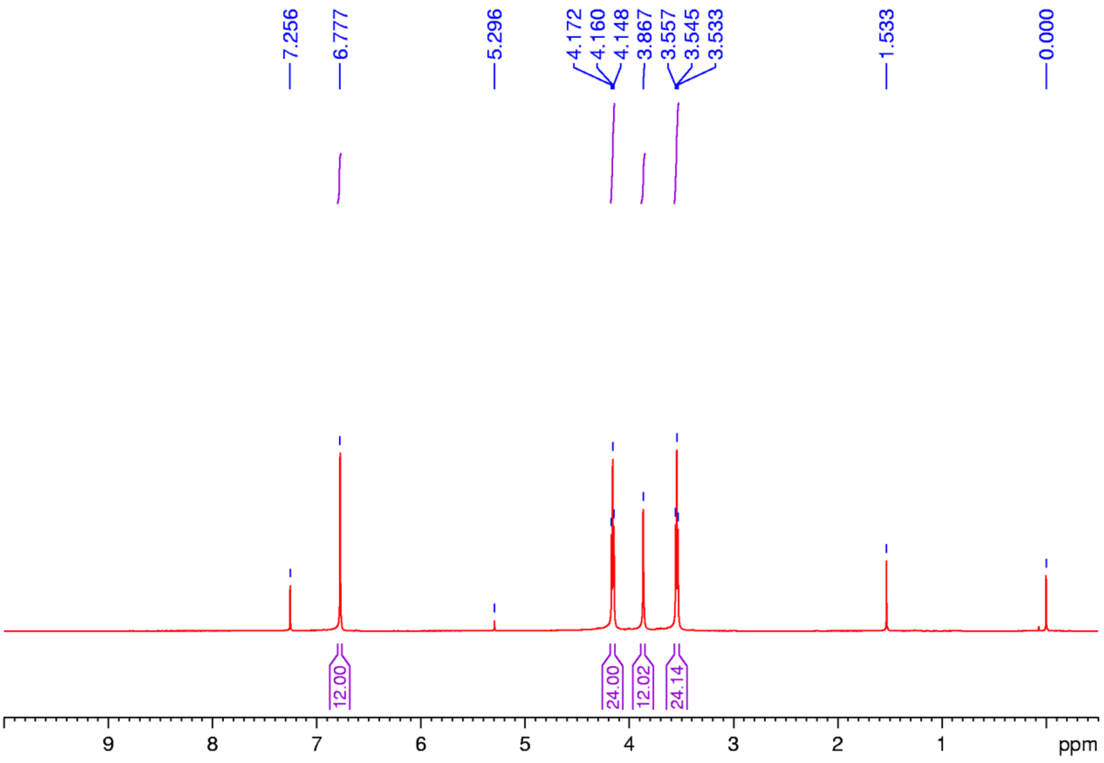

Figure S5. ^{1}H NMR spectrum (400 MHz, $CDCl_3$, 298 K) of **4**.

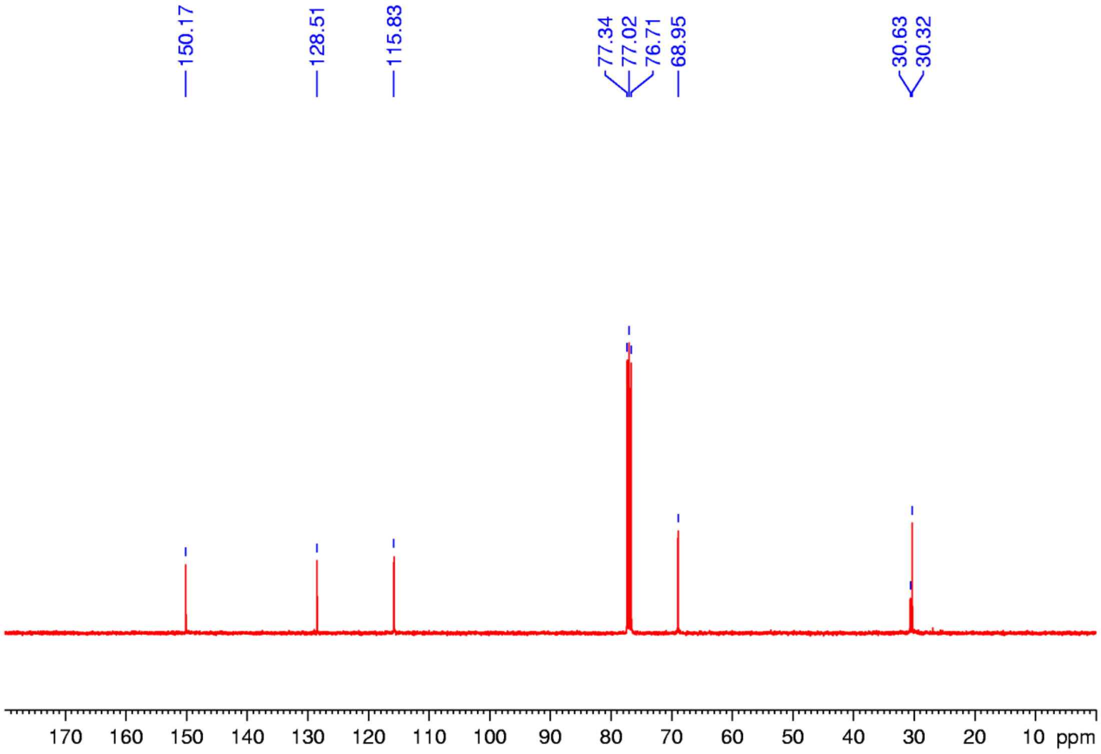

Figure S6. ^{13}C NMR spectrum (100 MHz, $CDCl_3$, 298 K) of **4**.

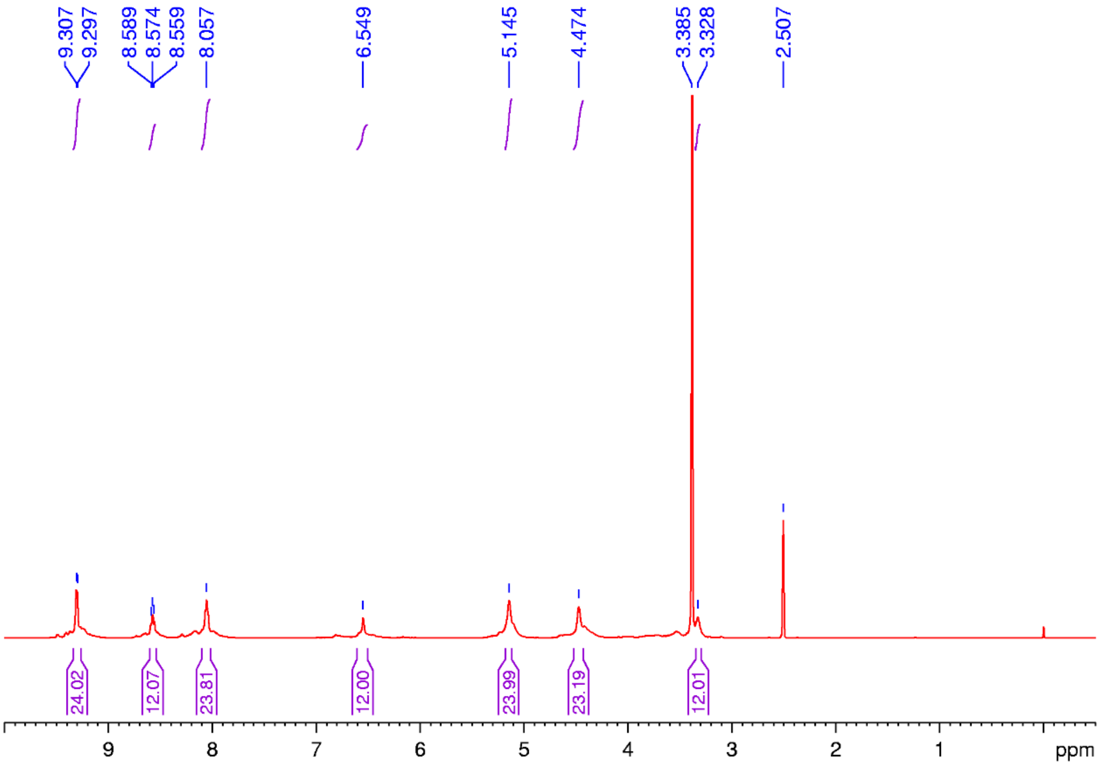

Figure S7. ^{1}H NMR spectrum (500 MHz, DMSO-*d6*, 298 K) of **PCP6**.

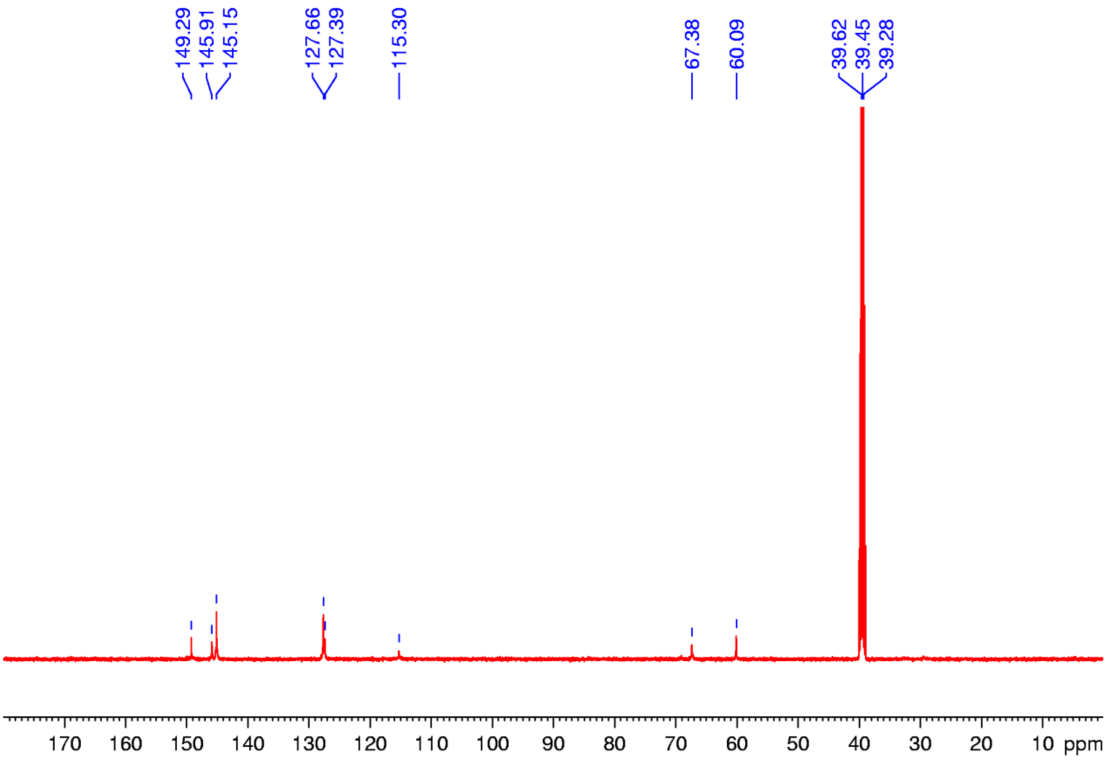

Figure S8. ^{13}C NMR spectrum (125 MHz, DMSO-*d6*, 298 K) of **PCP6**.

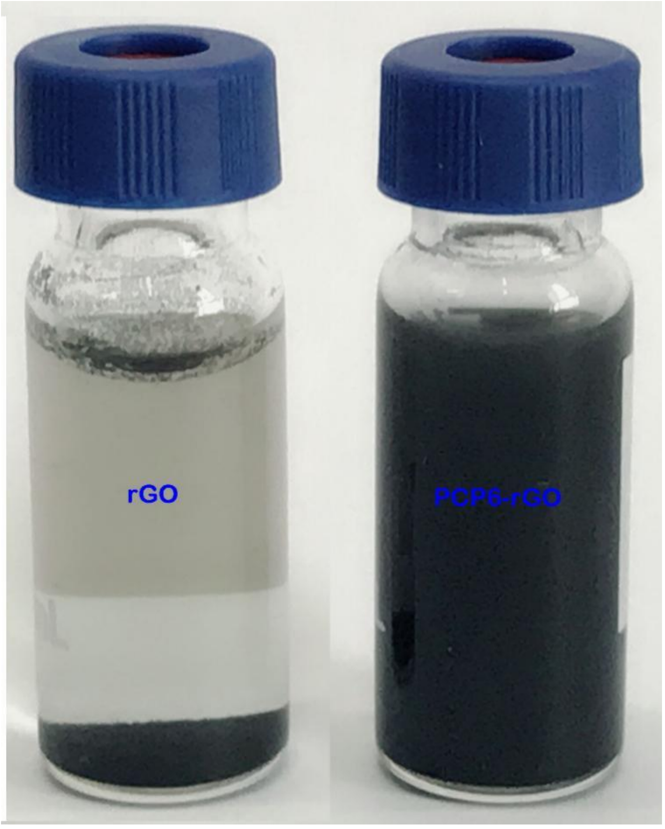

Figure S9. The photographs of rGO, PCP6-rGO aqueous dispersion.

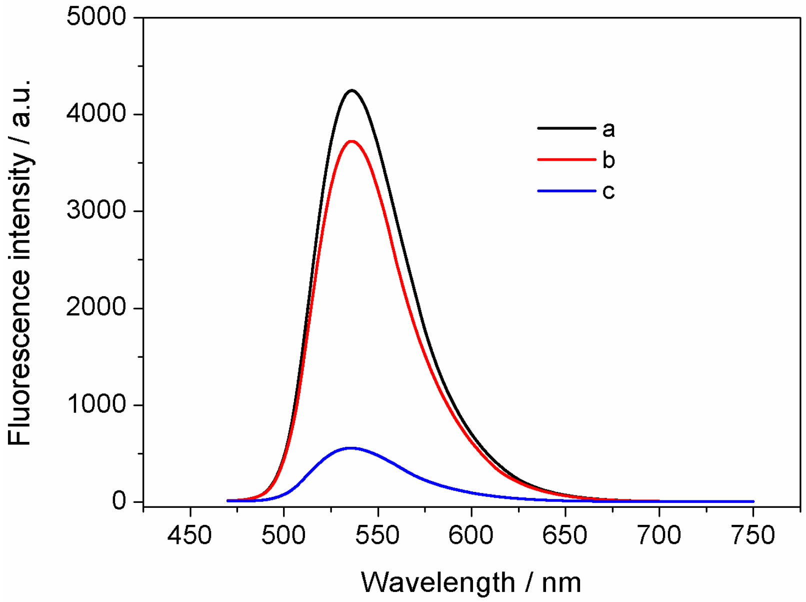

Figure S10. Fluorescence spectra of 10 μM AO (a), 10 μM AO in the presence of 20 μM PCP6 (b), and 10 μM AO in the presence of 18 μg mL^{-1} PCP6-rGO (c).

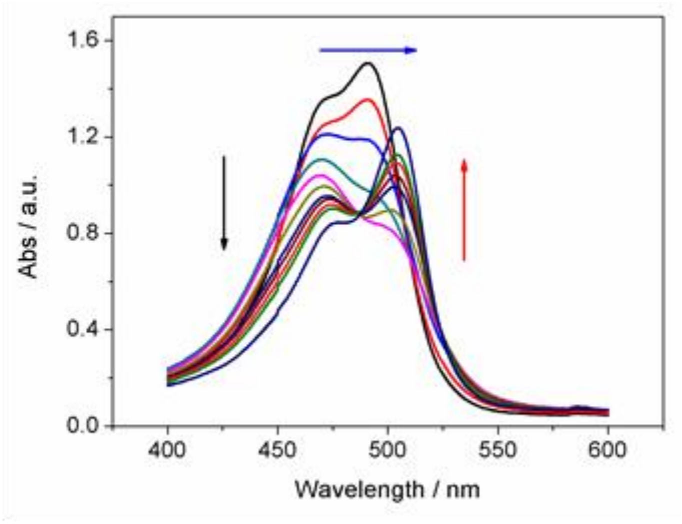

Figure S11. UV–vis spectra of 10 μM AO in the presence of 0, 5.0, 10.0, 15.0, 20.0, 25.0, 30.0, 35.0, 40.0, 45.0, and 50.0 μM of PCP6 in PBS (pH = 7.2).

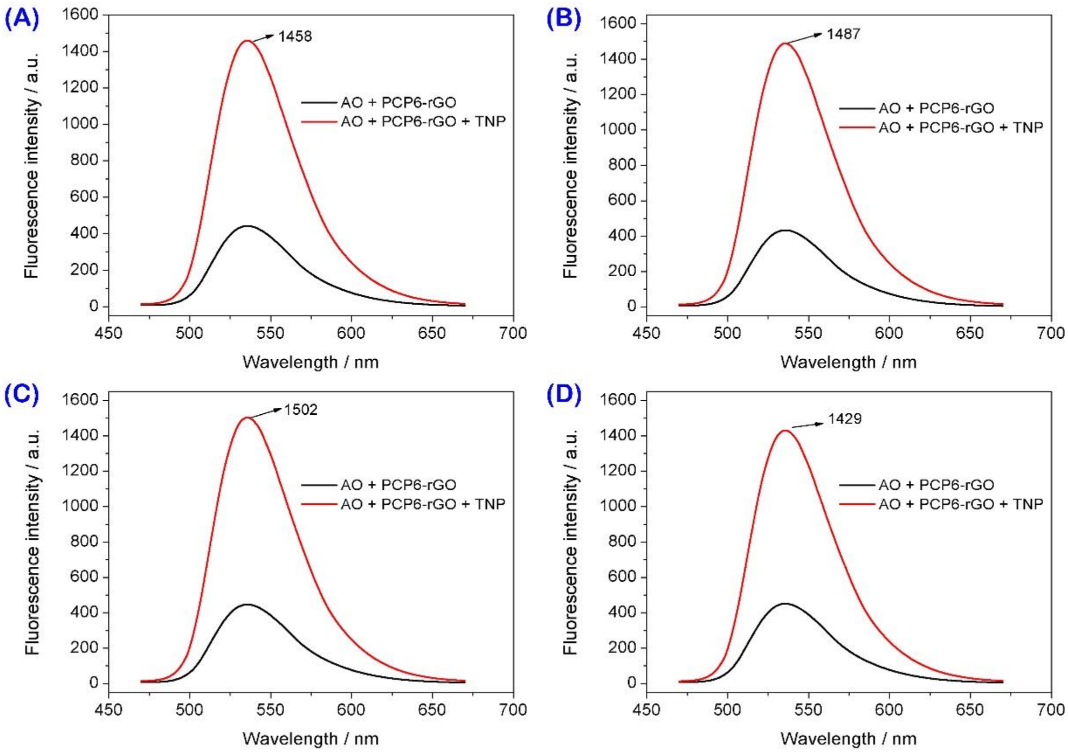

Figure S12. The effect of different temperatures on the recovering performance of TNP towards AO@PCP6-rGO; the same concentration of TNP with 20 μM and the temperature at 25 (A), 35 (B), 45 (C), and 50 ^{0}C (D), respectively.

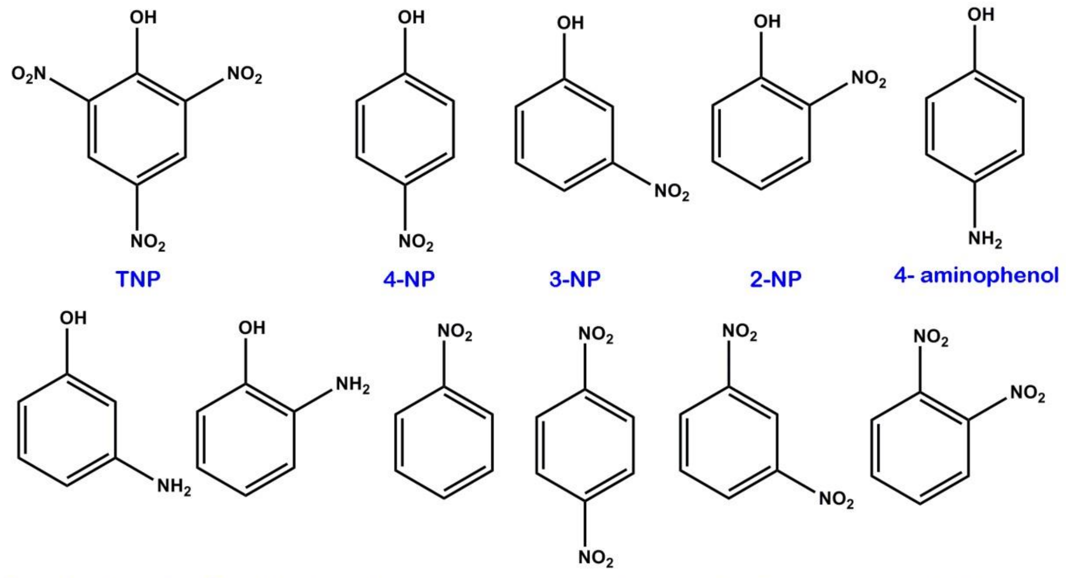

Figure S13. The chemical structures of TNP, 4-NP, 3-NP, 2-NP, 4-aminophenol, 3-aminophenol, 2-aminophenol, NB, 1,4-NB, 1,3-NB and 1,2-NB, respectively.

Table S1 Comparison of different methods for quantitative detection of TNP.

Electrode or matrix	Method	Liner range (μM)	LOD (μM)	Ref
Tb-CDs	Fluorescence	0.5–100	0.2	[4]
Ag nanoclusters	Fluorescence	0.1-20	0.1	[5]
GQDs	Fluorescence	0.1-15	0.09	[6]
MoS_2-QDs	Fluorescence	0.099-36.5	0.095	[7]
BNQDs	Fluorescence	0.25-200	0.14	[8]
N-GQDs	Fluorescence	1-60	0.3	[9]
Cu-BTC/ERGO/GCE	DPV	0.2-10	0.1	[10]
PCP6-rGO	Fluorescence	0.01-125	0.0035	This work

Reference

1. Chen, W.; Zhang, Y.Y.; Li, J.; Lou, X.B.; Yu, Y.H.; Jia, X.H.; Li, C.J. Synthesis of a cationic water-soluble pillar[6]arene and its effective complexation towards naphthalenesulfonate guests. *Chem. Commun*. **2013**, *49*, 7956–7958.

2. Yu, G.C.; Zhou, J.; Shen, J.; Tang, G.P.; Huang, F.H. Cationic pillar[6]arene/ATP host–guest recognition: selectivity, inhibition of ATP hydrolysis, and application in multidrug resistance treatment. *Chem. Sci*. **2016**, *7*, 4073–4078.
3. Wu, J.R.; Mu, A.; Li, B.; Wang, C.Y.; Fang, L.; Yang, Y.W. Desymmetrized Leaning Pillar[6]arene. *Angew. Chem*. **2018**, *130*, 1–7.
4. Chen, B.B.; Liu, Z.X.; Zou, H.Y.; Huang, C.Z. Highly selective detection of 2,4,6-trinitrophenol by using newly developed terbium-doped blue carbon dots. Analyst **2016**, *141*, 2676–2681.
5. Zhang, J.R.; Yue, Y.Y.; Luo, H.Q.; Li, N.B. Supersensitive and selective detection of picric acid explosive by fluorescent Ag nanoclusters. Analyst **2016**, *141*, 1091–1097.
6. Li, Z.; Wang, Y.; Li, Y.N.; Kokot, S. A sensor based on blue luminescent graphene quantum dots for analysis of a common explosive substance and an industrial intermediate 2,4,6-trinitrophenol. *Spectrochimica Acta Part A: Molecular and Biomolecular Spectroscopy* **2015**, *137*, 1213–1221.
7. Wang, Y.; Ni, Y.N. Molybdenum disulfide quantum dots as a photoluminescence sensing platform for 2,4,6-trinitrophenol detection. *Anal. Chem*. **2014**, *86*, 7463−7470.
8. Peng, D.; Zhang, L.; Li, F.F.; Cui, W.R.; Liang, R.P.; Qiu, J.D. Facile and green approach to the synthesis of boron nitride quantum dots for 2,4,6-trinitrophenol sensing. *ACS Appl. Mater. Interfaces* **2018**, *10*, 7315−7323.
9. Lin, L.P.; Rong, M.C.; Lu, S.S.; Song, X.H.; Zhong, Y.X.; Yan, J.W.; Wang, Y.R.; Chen, X. A facile synthesis of highly luminescent nitrogen-doped graphene quantum dots for the detection of 2,4,6-trinitrophenol in aqueous solution. Nanoscale **2015**, *7*, 1872–1878.
10. Wang, Y.; Cao, W.; Wang, L.Y.; Zhuang, Q.F.; Ni, Y.N. Electrochemical determination of 2,4,6-trinitrophenol using a hybrid film composed of a copper-based metal organic framework and electroreduced graphene oxide. *Microchimica Acta* **2018**, *185*, 315-323.
